# The effects of receiving diet roughage inclusion on performance, health, and serum metabolite characteristics of newly received beef calves

**DOI:** 10.1093/tas/txad039

**Published:** 2023-04-04

**Authors:** Colton A Robison, Kaitlyn N Pierce, Ryan R Reuter, Andrea L Warner, Blake K Wilson

**Affiliations:** Department of Animal and Food Sciences, Oklahoma State University, Stillwater, OK 74078, USA; Department of Animal and Food Sciences, Oklahoma State University, Stillwater, OK 74078, USA; Department of Animal and Food Sciences, Oklahoma State University, Stillwater, OK 74078, USA; Department of Animal and Food Sciences, Oklahoma State University, Stillwater, OK 74078, USA; Department of Animal and Food Sciences, Oklahoma State University, Stillwater, OK 74078, USA

**Keywords:** bovine respiratory disease, dietary roughage, energy density, feedlot, receiving

## Abstract

Current dogma suggests increased dietary roughage may improve calf health at the expense of performance during receiving. In experiment 1, the effects of increasing dietary roughage on performance and clinical health of high-risk heifers was evaluated over a 56-d receiving period. Heifers (*n* = 589; initial body weight; **BW** = 230 ± 33 kg) were sourced from Oklahoma livestock auctions from April through October of 2019. Heifers were randomly assigned to pens, which were randomly assigned to 1 of 3 experimental dietary treatments in a randomized complete block design. Diets contained either: 1) 15% roughage (**R15**), 2) 30% roughage (**R30**), or 3) 45% roughage (**R45**) in the form of prairie hay. Orthogonal contrasts were used to test for linear and quadratic responses among experimental treatments. There was a linear decrease in overall average daily gain (**ADG**; *P* ≤ 0.0001) with increasing roughage inclusion which resulted in a linear decrease (*P* ≤ 0.0001) in heifer final BW. A linear increase (*P* ≤ 0.01) was observed for overall dry matter intake (**DMI**), and overall gain:feed (**G:F**) decreased linearly (*P* ≤ 0.0001) as dietary roughage concentration increased. A quadratic response to decreasing roughage was observed (*P* = 0.02) for the percent of calves treated three times for bovine respiratory disease (**BRD**). No other responses (*P* ≥ 0.11) were detected in animal health variables. In experiment 2, Angus steers (*n* = 12) and heifers (*n* = 6; BW = 272 ± 28 kg) were acquired from a single ranch in Oklahoma to evaluate the same experimental dietary treatments on serum metabolite concentrations. Animals were randomly assigned to experimental treatments, with animal serving as the experimental unit in experiment 2. Statistical models for serum metabolites in experiment 2 were analyzed using repeated measures with the effects of treatment, time, and treatment × time. In experiment 2, there were tendencies for treatment × time interactions for blood urea nitrogen (**BUN**; *P* = 0.07) and nonesterified fatty acid (**NEFA**; *P* = 0.06) concentrations. No metabolites were affected by treatment (*P* ≥ 0.11), while all metabolites were impacted by time (*P* ≤ 0.02). In summary, growth performance was improved in calves as dietary roughage concentration decreased with minimal impacts on health and serum metabolites. These results suggest that diets containing as little as 15% roughage can be used during receiving to improve calf performance without compromising calf health when fibrous byproducts are included in the diet.

## INTRODUCTION

Newly received calves often arrive at feedlots in a catabolic state induced by the sustained feed and water deprivation that animals endure during the marketing and transport processes (Richeson et al., 2019). The resulting negative energy balance, combined with traditionally low DMI postarrival frequently observed in newly received calves, can impair immune function, which may lead to decreased performance and greater occurrence of health-related issues due to bovine respiratory disease (BRD; Wilson et al., 2017; Richeson et al., 2019). Thus, proper nutritional management of newly received calves is paramount to allow for recovery from the stresses associated with marketing and transport (Krehbiel, 2011; Wilson et al., 2017).

Roughage level and energy density in receiving diets have been investigated by researchers and debated among nutritionists for decades (Lofgreen et al., 1975). Current dogma with regard to receiving calf nutrition is that performance and efficiency increase as roughage level in receiving diets decreases, but that this performance increase comes at the expense of increased morbidity (Richeson et al., 2019). Samuelson et al. (2016) reported that nutritionists formulate receiving diets with roughage concentrations greater than 40% on average, and frequently incorporate grain at 30% to 40% of receiving diets. Lofgreen et al. (1975) reported that increasing diet energy density improved performance and also concluded that morbidity may increase with increasing levels of dietary energy. A meta-analysis completed by Rivera et al. (2005) concluded that morbidity caused by BRD slightly increased as roughage inclusion decreased. The authors suggested that providing diets containing 50% to 75% concentrate was optimum for starting light-weight, stressed, receiving cattle. Alternatively, Berry et al. (2004) reported no differences in morbidity among calves offered diets differing in diet energy and starch concentrations.

Historical research supporting the recommendations regarding roughage and energy density in receiving diets lacks the inclusion of fibrous byproducts that commonly contribute a large percentage of the diet offered to receiving cattle U.S. feedlots today. Fibrous byproducts provide an opportunity to increase diet energy density without increasing dietary starch concentration (Richeson et al., 2019). Therefore, the objective of the current experiment was to determine the effect of roughage inclusion and subsequent differences in diet energy density on receiving calf performance, clinical health, and metabolite characteristics of newly received beef calves. We hypothesized that calf performance would increase, and the number of calves treated for BRD would decrease slightly with decreasing dietary roughage inclusion.

## MATERIALS AND METHODS

All live animal procedures for the current experiments were approved by the Oklahoma State University Institutional Animal Care and Use Committee (Animal Care and Use Protocol number: AG-19-8). These experiments were conducted at the Willard Sparks Beef Research Center (WSBRC) located in Stillwater, OK.

### Cattle Background and Arrival Processing

In experiment 1, a total of 589 crossbred beef heifers (body weight; BW = 230 ± 33 kg) were purchased at livestock auctions throughout Oklahoma and sent to an order buyer facility in central Oklahoma. All heifers remained at the order buyer facility for at least 4 d (range = 4 to 7 d) before being transported for approximately 1.5 h to the WSBRC on April 4, 2019 (Block 1; *N* = 62; 3 pens), May 13, 2019 (Block 2; *n* = 73; 3 pens), June 10, 2019 (Block 3; *n* = 60; 3 pens), July 22, 2019 (Block 4; *n* = 80; 3 pens), August 19, 2019 (Block 5; *n* = 179; 6 pens), October 9, 2019 (Block 6; *n* = 69; 3 pens), and October 23, 2019 (Block 7; *n* = 66; 3 pens). Immediately upon arrival to the WSBRC (day −1), calves were individually weighed on validated individual scales (Avery Weigh-Tronix; Fairmont, MN) and administered individual identification tags. Calves were then allowed to rest for 12 to 24 h in holding pens and provided ad libitum access to prairie hay and water before initial processing on day 0.

In experiment 2, a total of 18 Angus steers (*n* = 12) and heifers (*n* = 6; BW = 272 ± 28 kg) were acquired from a single ranch and transported approximately 48 km to Stillwater, OK on December 17, 2019. Similar to the heifers in experiment 1, calves in experiment 2 were individually weighed and administered individual identification tags on day −1 and were allowed 12 to 24 h of rest in a common holding pen with ad libitum access to prairie hay and water before initial processing on day 0.

At initial processing (day 0), calves enrolled in experiments 1 and 2 were weighed, vaccinated against clostridial pathogens *Clostridium chauvoei*, *C. novyi*, *C. perfringens* C and D, *C. septicum, C. sordellii,* and *Moraxella bovis* (Vision 7 20/20 with SPUR; Merck Animal Health, Madison, NJ) and against viral bovine herpesvirus-1, bovine viral diarrhea virus type 1 and 2, parainfluenza-3 virus, and bovine respiratory syncytial virus (Titanium 5; Elanco Animal Health, Greenfield, IN). Calves were also administered a *Mannheimia haemolytica* bacterin (Nuplura; Elanco Animal Health), drenched with an oral anthelmintic (Safeguard; Merck Animal Health), and an insecticide (Standguard; Elanco Animal Health) was applied topically.

Heifers from experiments 1 and 2 were administered a growth implant containing 80 mg of trenbolone acetate, 8 mg of estradiol, and 29 mg of tylosin tartrate (Component TE-IH; Elanco Animal Health), while steers in experiment 2 were administered a growth implant containing 80 mg of trenbolone acetate, 16 mg of estradiol, and 29 mg of tylosin tartrate (Component TE-IS; Elanco Animal Health). Calves were sorted upon exiting the chute and were immediately allotted to assigned receiving pens following processing on day 0.

### Experimental Treatments and Receiving Management

Experimental dietary treatments (Table 1; DM basis) consisted of 1) a low roughage (**R15**) diet (15.0% prairie hay, 46.5% Sweet Bran (branded wet corn gluten feed-based product; Cargill Inc., Dalhart, TX), 32.5% rolled corn, and 6% dry supplement), 2) an intermediary roughage (**R30**) diet (30.0% prairie hay, 39.0% Sweet Bran (Cargill Inc.), 25% rolled corn, and 6% dry supplement), 3) or a high roughage (**R45**) diet (45.0% prairie hay, 31.5% Sweet Bran (Cargill Inc.), 25.0% rolled corn, and 6.0% dry supplement). All prairie hay was ground through a commercial hay grinder using a 17.8-cm screen to target a maximum roughage particle length of 17.8 cm. Experimental treatment diets were formulated to meet or exceed NASEM (2016) nutrient requirements.

**Table 1.** Ingredient and nutrient composition of experimental diets^1^

	Experimental dietary treatment^2^		
Ingredient, % of DM	R15	R30	R45
Rolled corn	32.50	25.00	17.50
Prairie hay	15.00	30.00	45.00
Sweet Bran^3^	46.50	39.00	31.50
Dry supplement^4^	6.00	6.00	6.00
Nutrient composition, DM basis			
Dry matter, %	71.59	73.02	74.23
Crude protein, %	16.94	15.96	14.98
Acid detergent fiber, %	18.10	22.60	28.93
peNDF^5^, %	23.38	29.74	36.11
TDN^6^, %	70.70	63.88	60.35
NE_m_^7^, Mcal/kg	1.47	1.26	1.15
NE_g_^7^, Mcal/kg	0.88	0.69	0.59
Ca, %	0.71	0.85	0.74
P, %	0.65	0.58	0.50
K, %	1.01	0.96	0.93
Mg, %	0.30	0.31	0.30

^1^Diet analyses were performed by Servi-Tech Laboratories; Dodge City, KS.

^2^Treatments included (DM basis): R15 =** **15% prairie hay, 46.50% Sweet Bran, 32.50% rolled corn, 6% dry supplement; R30 =** **30% prairie hay, 39% Sweet Bran, 25% rolled corn, 6% dry supplement; R45 =** **45% prairie hay, 31.50% Sweet Bran, 17.50% rolled corn, 6% dry supplement.

^3^Sweet Bran (branded wet corn gluten feed-based product; Cargill Inc.).

^4^Dry supplement was formulated to contain (% DM basis) 40.0% ground corn, 29.6% limestone, 20.0% wheat middlings, 7.0% urea, 1.0 % salt, 0.53% magnesium oxide, 0.51% zinc sulfate, 0.17% manganese oxide, 0.13% copper sulfate, 0.08% selenium premix (0.6%), 0.0037% cobalt carbonate, 0.32% vitamin A (30,000 IU/g), 0.10% vitamin E (500 IU/g), 0.009% vitamin D (30,000 IU/g), 0.20 % tylosin (Tylan-40, Elanco Animal Health, Greenfield IN), and 0.33% monensin (Rumensin- 90; Elanco Animal Health).

^5^Physically effective fiber proved by the prairie hay and Sweet Bran in the diet.

^6^Calculated according to Weiss et al. (1992).

^7^Calculated according to NASEM (2016).

Pen was considered the experimental unit in experiment 1 and animal was considered the experimental unit in experiment 2. Calves were stratified by BW within block and were randomly assigned to 1 of 3 experimental pens in both experiments. Experimental treatments were previously randomly assigned to receiving pen. A total of 27 receiving pens were used for experiment 1, with 9 replications per treatment. A total of three receiving pens were used for experiment 2, with 4 steers and 2 heifers per pen.

All animals were housed in 12.5 × 30.5-m soil-surfaced, open-air receiving pens that contained continuous flow concrete watering tanks (Model J 360-F; Johnson Concrete, Hastings, NE) and 12.2-m concrete bunks, which provided approximately 46 cm of linear bunk space per animal for heifers in experiment 1 and 152 cm of bunk space per animal in experiment 2. Calves received ad libitum access to water and experimental diets throughout the duration of 56-d receiving experiment. Long-stem hay was provided at 0.91 kg per head daily in the bunk for the first 4 d of the experiment. Feed bunks were read at 0530 and 1730 hours and feed was mixed and delivered once daily at 0800 hours by a trailer-mounted feed mixer (274-12B; Roto-Mix, Dodge City, KS). Feed calls were adjusted daily to target minimal orts (crumbs) being left in the bunk, and feed refusals were collected daily at 0600 hours. Daily residual feed and feed refusals weighed before BW collections were dried for dry matter (DM) determination and the resulting feed DM was removed from the DM feed delivered for dry matter intake (DMI) calculations.

Ration samples were collected twice per week. Ration samples and feed refusals were dried in a forced air oven for 48 h at 60 °C for DM determination. Weekly ration samples were composited by month and analyzed at a commercial laboratory (Servi-Tech Inc., Dodge City, KS) for determination of nutrient composition. Total digestible nutrients (TDN) were calculated as described by Weiss et al. (1992). Net energy for maintenance (NE_m_) and gain (NE_g_) were calculated using equations provided by NASEM (2016).

Particle size of prairie hay and Sweet Bran (Cargill Inc.) were determined with a 3.8 L sample using a three-sieve forage particle separator (Nasco; Fort Atkinson, WI). The sieves were shaken in one direction five times, rotated one quarter turn and repeated for a total of 8 sets or 40 shakes. The physically effective neutral detergent fiber (peNDF) for prairie hay and Sweet Bran was estimated by calculating the percent of the sample remaining in the top three sieves and multiplying by the NDF content of the feedstuff. To determine the peNDF from the roughage and Sweet Bran of each diet, the peNDF of each contributing ingredient was multiplied by the percent inclusion in the diet. The respective peNDF values were then added to determine total peNDF for the diet.

### Evaluation of BRD for Antimicrobial Treatments

Calves were evaluated daily by two trained personnel for the detection of clinical signs indicative of (or consistent with) BRD. Calves were assigned severity scores (SSs) based upon the DART system with modifications described by Wilson et al. (2016) and Step et al. (2008). Numerical SS ranged from 0 to 4. An SS of 0 indicated an animal was clinically normal, an SS of 1 indicated mild clinical signs, an SS of 2 indicated an animal exhibiting moderate clinical signs, an SS of 3 indicated severe clinical signs, and an SS of 4 would indicate a moribund animal that required immediate attention. Animals that received an SS of 1 or greater were eligible to be taken to the processing chute for rectal temperature measurement (GLM-500, GLA Agricultural Electronics, San Luis Obispo, CA). Animals that received a subjective SS of 1 or 2 and had a rectal temperature greater than or equal to 40 °C were eligible for antimicrobial treatment. Calves that were assigned an SS of 3 or 4 received antimicrobial treatment regardless of rectal temperature. All calves regardless of whether or not antimicrobial treatment was administered were immediately returned to a home pen from the processing chute.

All antimicrobials were administered subcutaneously according to the manufacturer’s label and Beef Quality Assurance guidelines (NCBA, 2001). Calves were eligible to receive an antimicrobial up to three times on alternating sides of the animal. If an animal did not respond to antimicrobial treatment and continued to receive a SS score of 1 or greater after the third antimicrobial administration post treatment interval had passed or became unable to compete in the pen, the animal was considered ‘chronic’ and removed from the experiment. Antimicrobial treatment with tilmicosin phosphate (Micotil; 10 mg/kg, Elanco Animal Health) was administered the first time BRD treatment criteria were met. Following a moratorium of 120 h after tilmicosin administration, florfenicol (Nuflor; 40 mg/kg, Merck Animal Health) was administered if antimicrobial treatment criteria for BRD were met a second time. If BRD treatment criteria were met for the third time following a florfenicol moratorium of 72 h, ceftiofur crystalline free acid (Excede; 6.6 mg/kg, Zoetis) was administered.

### Data Collection

Individual BW was recorded for all heifers in experiment 1 on days 0, 14, 28, 42, and 56. BW was recorded before feeding at approximately 0530 hours with no feed or water withdrawal. All BW were adjusted using a calculated 2% pencil shrink (BW × 0.98). Individual BW were averaged within pen to calculate an average BW for the pen. Average daily gain (ADG) was calculated for each animal by dividing the individual BW gained during the period by the number of days on feed during the period. Pen ADG was calculated by averaging individual ADG for each animal in a pen for that period. DMI was calculated within period by taking the total weight of DM fed divided by the number of calves and the days on feed for that period. Gain to feed ratio (G:F) was calculated within period by dividing pen average ADG by the pen average DMI for each respective period.

Performance and feed data for animals that died or were removed from the experiment were excluded from statistical analyses. Intake data were corrected for mortalities and removals by removing an animal’s daily DMI at the average of the pen DMI from the appropriate pen if the animal was still gaining BW at the time of death or removal. If an animal was not gaining BW at the time of removal, DMI for that animal was removed at the average of the pen DMI as previously described until the animal ceased gaining BW and then removed at calculated maintenance intake from the time the animal began to lose weight until the removal or mortality date. Maintenance intake was calculated using the NASEM (2016) net energy maintenance equation NE_m_ = 0.077 × (SBW)^0.75^ and the respective experimental treatment diet NE_m_ concentration.

All animals in experiment 2 were bled via jugular venipuncture using 10 mL serum vacutainer tubes (BD Vacutainer; Thermo Fisher Scientific, Waltham, MA) to evaluate the metabolite responses of glucose, L-lactate, nonesterified fatty acids (NEFA), and blood urea nitrogen (BUN) to dietary roughage treatments. Blood collections occurred on h 0 (immediately prior to feeding experimental dietary treatments for the first time), on day 1 (hour 4; 4 h after feeding experimental dietary treatments for the first time), and on days 2, 3, 4, 5, 6, 13, and 20. For all days (except the hour 4 timepoint) blood was collected at the same time (0600 hours; immediately prior to feeding). Following blood collection, serum was harvested by centrifuging the whole blood at 1,294 × *g* for 10 min at 4 °C (Sorvall RC6; Thermo Scientific, Waltham, MA). Serum was then stored at −80 °C until metabolite analyses could be conducted.

Serum samples were thawed immediately prior to glucose, lactate, NEFA, and BUN analysis. Glucose and lactate were analyzed using an immobilized enzyme system (YSI Model 2950 D; YSI Inc., Yellow Springs, OH). BUN was analyzed in duplicate utilizing the methods described by Marsh et al. (1965) adapted for a 96-well plate. Briefly, a ferric chloride-phosphoric acid solution and 20% sulfuric acid were used to prepare a BUN acid solution. Thiosemicarbazide and diacetyl (2,3-butanedione) monoxime were used to prepare the BUN color reagent. Samples were then analyzed against a standard curve on a microplate reader (Biotek EPOCH, Biotek Instruments Inc., Winooski, VT) at 520 nanometers (nm). Serum was analyzed for NEFA concentration by use of a standard NEFA quantitation kit (NEFA-C Kit; WAKO Chemicals USA, Richmond, VA) according to manufacturer’s instructions based on the acyl-CoA synthetase-acyl-CoA oxidase method. Samples were analyzed in duplicate in 96-well plates on the same microplate reader (Biotek EPOCH, Biotek Instruments Inc.) at 550 nm.

### Statistical Analysis

Data from both experiments were analyzed using the MIXED procedure of SAS 9.4 (SAS Institute Inc., Cary, NC). In experiment 1, experimental treatments were arranged in a generalized randomized complete block design (*n* = 7 blocks) with 9 pen replications per treatment. Pen served as the experimental unit for all dependent variables. Treatment was included in the model as the fixed effect and block served as a random effect. Linear and quadratic contrasts were performed for the main effects for the three experimental treatments (R15, R30, and R45). All performance and intake data from calves removed in the experiment were excluded from the statistical analysis (deads and removals out data) with the exception of the health variables.

In experiment 2, animal served as the experimental unit for all serum metabolite data. Metabolite data were first assessed for normality using the Shapiro–Wilk test statistic in the UNIVARIATE procedure of SAS 9.4. Lactate and NEFA concentrations were determined to be nonnormal and were logarithm (base 10) transformed to achieve normality. A repeated measures analysis was then used where the fixed effects of treatment, time, and the resulting treatment × time interaction were included in the model. The covariance structure with the lowest Akaike information criterion was used for each variable. For both experiments, results were considered significant where *P* ≤ 0.05, and tendencies were considered where 0.05 > *P *≤ 0.10.

## RESULTS AND DISCUSSION

### Animal Performance (experiment 1)

Performance data are presented in Table 2. As expected, there was no difference in BW (*P* ≥ 0.38) on day 0. There were also no differences (*P* ≥ 0.13) observed in BW between treatments on day 14. However, there was a linear increase (*P* ≤ 0.01) in BW on days 28, 42, and 56 with decreasing dietary roughage concentration. The lack of difference in BW on day 14 may have been the result of lower initial feed intake and thus overall energy intake due to the stressful transition to the feedlot and associated greater morbidity within first few days following arrival. DMI was 2.2%, 2.1%, and 2.2% of BW during the first 14 d for the R15, R30, and R45 treatment groups, respectively. It has been reported that calves often only consume 0.5% to 1.5% of their BW during the first 2 wk after arrival to the feedlot (Krehbiel et al., 2011; Richeson et al., 2019). ADG increased linearly with decreasing dietary roughage concentration during the first three periods (*P* ≤ 0.05) and also increased linearly throughout the entire experiment (days 0 to 56; *P* ≤ 0.0001). Interestingly, no response in ADG was observed from days 42 to 56 (*P* ≥ 0.18).

**Table 2.** Effect of roughage inclusion in receiving diets on growth performance, intake, feed efficiency in newly received high-risk heifers (experiment 1)

	Experimental dietary treatment^1^				Contrasts	
Item	R15	R30	R45	SEM^2^	Linear	Quadratic
BW^3^, kg						
d 0	225	225	226	7.9	0.26	0.38
d 14	244	243	241	8.8	0.13	0.95
d 28	267	263	259	9.5	<0.01	0.89
d 42	288	280	272	8.3	<0.0001	0.99
d 56	309	301	291	9.8	<0.0001	0.69
ADG^4^, kg						
d 0 to 14	1.38	1.28	1.11	0.134	0.04	0.73
d 14 to 28	1.59	1.45	1.29	0.114	0.05	0.94
d 28 to 42	1.50	1.17	0.89	0.160	<0.0001	0.84
d 42 to 56	1.56	1.54	1.40	0.140	0.18	0.51
d 0 to 56	1.51	1.36	1.17	0.058	<0.0001	0.53
DMI^5^, kg						
d 0 to 14	4.94	4.77	5.02	0.212	0.54	0.08
d 14 to 28	7.60	7.65	7.82	0.348	0.24	0.70
d 28 to 42	8.54	8.81	9.15	0.455	0.04	0.89
d 42 to 56	8.82	9.45	10.36	0.477	<0.001	0.60
d 0 to 56	7.45	7.62	8.07	0.346	<0.01	0.41
G:F^6^						
d 0 to 14	0.279	0.275	0.223	0.0298	0.04	0.28
d 14 to 28	0.212	0.190	0.166	0.0144	0.03	0.96
d 28 to 42	0.176	0.134	0.099	0.0187	<0.0001	0.74
d 42 to 56	0.176	0.163	0.133	0.0118	<0.001	0.31
d 0 to 56	0.204	0.180	0.146	0.0075	<0.0001	0.24

^1^Treatments included (DM basis): R15 =** **15% prairie hay, 46.50% Sweet Bran, 32.50% rolled corn, 6% dry supplement; R30 =** **30% prairie hay, 39% Sweet Bran, 25% rolled corn, 6% dry supplement; R45 =** **45% prairie hay, 31.50% Sweet Bran, 17.50% rolled corn, 6% dry supplement.

^2^ *n* = 9 pens per treatment.

^3^Body weight was adjusted using a 2% calculated shrink.

^4^Pen ADG was calculated from individual shrunk BW gain, kg divided by days on feed for each period.

^5^Pen DMI was calculated from total DMI for the pen for each period divided by the total steers and days on feed in each period.

^6^Pen G:F was calculated by dividing the ADG for the pen by the average daily DMI for the pen for each respective period.

A tendency for a quadratic response (*P* = 0.08) in DMI was detected from days 0 to 14, where calves on the R45 treatment tended to consume more feed than calves on the R30 treatment but did not differ from calves on the R15 treatment. There was no response (*P* ≥ 0.24) in DMI observed from day 14 to 28; however, DMI increased linearly (*P* ≤ 0.04) for the last two periods with increasing roughage in the diet. Overall DMI (*P* ≤ 0.01) throughout the experiment also increased linearly as roughage concentration increased. There was a linear decrease (*P* ≤ 0.04) in G:F with increasing dietary roughage across all time periods during the experiment.

The linear increase for BW and ADG as concentrate level within the diet increased is expected, as intake of dietary energy was greater for the lower roughage diets. The linear increase in DMI as roughage level increased can likely be explained by differences in caloric intake and physical ruminal capacity. Satiety in cattle consuming diets high in roughage may only be met when maximum ruminal capacity or ruminal fill is reached. Alternatively, lower levels of intake have been reported in diets with greater caloric density due to negative chemostatic feedback mechanisms (Krehbiel et al., 2006). Diets in the current experiment contained 0.88, 0.69, and 0.59 Mcals NE_g_/kg of DM for the R15, R30, and R45 diets, respectively; thus, the resulting average NE_g_ intake for each experimental group from days 0 to 56 would be 6.5, 5.3, and 4.8 Mcals NE_g_ per d, respectively.

The performance trend in the current experiment aligns similarly to that reported by Lofgreen et al. (1975), in which multiple experiments evaluated energy level in receiving diets offered to high-risk calves on performance and health characteristics. In their first experiment, Lofgreen et al. (1975) reported that BW gain was greater for the calves receiving the 1.10 Mcal NE_g_/kg diet (72% concentrate) than for the other two dietary treatments (0.84 and 1.01 Mcal NE_g_/kg; 20% and 55% concentrate, respectively). However, Lofgreen et al. (1975) also reported that DMI was directionally proportional to energy density in their first experiment where DMI increased as NE_g_ increased from 0.84 to 1.01 or 1.10, which is in disagreement with the results of the current experiment. Conversely, in their second experiment, DMI of receiving calves decreased as Lofgreen et al. (1975) replaced the 1.01 Mcal NE_g_/kg diet (55% concentrate) with a 1.19 Mcal NE_g_/kg diet (90% concentrate), which would align similarly with results from the current experiment.

Berry et al. (2004) reported that performance, intake, and G:F were unaffected when diet starch concentration was increased from 18.8% to 26.9%. These authors also reported that energy density (0.85 vs. 1.07 Mcal/kg NE_g_) had no effect on animal performance during a 42-d trial; however, a greater diet energy density in their experiment reduced overall experimental DMI (Berry et al., 2004). Research published by Fluharty and Loerch (1996) examined diets containing 1.15, 1.21, 1.25, or 1.30 Mcal NEg/kg and suggested that DMI and ADG increase as percent concentrate in the ration increases. In a review of dietary roughage concentration (diets ranging from 23.62% to 100% roughage) conducted by Rivera et al. (2005), the authors reported that ADG and DMI were negatively affected by increasing dietary roughage concentration, which partially agrees with results from the current experiment, as results from the current experiment suggest that DMI increases proportionally with increasing roughage level while ADG decreases proportionally with increasing roughage level.

While dietary roughage (15% to 45%) and concentrate (55% to 85%) levels in the current experiment are similar to many of other experiments discussed, it should be noted that diet composition in the current experiment may contrast in ingredient composition, roughage source (prairie hay vs. alfalfa hay or silage, etc.), grain (starch) content, and overall energy concentration (NEg) with the other experiments mentioned. Perhaps most importantly, diets in the current experiment provided the concentrate within diet via a combination of corn (starch) and Sweet Bran, a branded wet corn gluten feed-based product (fibrous byproduct). In combination, these differences in composition may provide some explanation for the discrepancy in DMI response among experiments.

While the mechanisms for controlling DMI in ruminant animals are well-understood in theory, these mechanisms are often over simplified. In the simplest terms, the intake of high roughage, low-energy diets is primarily controlled by physical/rumen fill while the intake of high concentrate, high-energy diets is primarily controlled by chemostatic feedback mechanisms. In mixed diets, where the energy content of the concentrate portion of the diet is diluted through the addition of roughage, an animal must consume more feed from a diet that is higher in roughage in order to obtain the same quantity of energy as an animal consuming a similar diet that is higher in concentrate and lower in roughage.

However, when physical fill is not truly limiting intake, as is likely the case in all the mixed diets discussed herein, animals fed a diet ever so slightly higher in roughage should be able to consume a small additional amount of DM and thus additional energy. In reality these mechanisms are not as simple as in theory and determining the increase in roughage level that allows for the animal to consume additional DM and thus additional energy while still having the physical and chemostatic capacity to do so is complicated. It is also likely that this intake mechanism is further complicated by the inherent variation within roughage sources commonly used in receiving diets (hays, silages, hulls, etc.). Thus, it is likely that the specific roughage level that influences the shift from physical to chemostatic intake control varies between roughage sources as well and is not strictly a function of roughage level. These factors may also help explain some of the intake differences observed between the current experiment and other experiments discussed herein.

While Berry et al. (2004) varied the proportion of dietary concentrate coming from cereal grain (starch) vs. fibrous byproduct sources within the diet (low and high energy as well as low and high starch), Fluharty and Loerch (1996) and Lofgreen et al. (1975) would not have included any fibrous byproducts in their diets. Diets in the current experiment contained 85%, 70%, and 55% concentrate for the R15, R30, and R45 diets, respectively with more of that concentrate, 46.5%, 39.0%, 31.5%, respectively, coming from Sweet Bran (fibrous byproduct) than from corn (starch), 32.5%, 25.0%, 17.5%, respectively. In contrast, the diets in Berry et al. (2004) ranged from 13.0% to 37.5% corn, the diets in Lofgreen et al. (1975) ranged from 2.5% to 49.5% barley (composition of the 90% concentrate diet was not provided), and the diets in Fluharty and Loerch (1996) ranged from 7.08% to 42.93% corn. Overall, there is greater variation in grain (starch) content within the diets for the other experiments discussed, but diets containing similar amounts of cereal grain to the current experiment were also reported in each of those experiments.

In addition to roughage and concentrate composition, the diets in the current experiment likely varied substantially from the other experiments mentioned in DM content. In the current experiment, the average diet DM was 72.9% (range = 71.59% to 74.23%), which can be primarily attributed to the increased inclusion of the Sweet Bran. Only Berry et al. (2004) indicated the diet DM of their experimental treatments, which averaged 89.4% DM; however, based on the dietary ingredients and respective levels in the diets, it can be speculated that diet DM from Lofgreen et al. (1975) would be greater than 85%, while the diet DM of Fluharty and Loerch (1996) would have varied substantially due to the inclusion of corn silage in some experimental diets.

Overall, performance data from the current experiment agree with literature previously reported such that animal performance (ADG and G:F) is increased as dietary energy density increases; however, few previously published experiments are in complete agreement with the intake results of the current experiment.

### Clinical Health (experiment 1)

A total of 32 heifers died (*n* = 17), were deemed ‘chronics’ (*n* = 7) or were otherwise removed from the experiment for non BRD related concerns (*n* = 8; coccidiosis, lameness, heat stress, etc.). Data not presented. Many of the ‘chronic’ animals ultimately had to be euthanized after removal from the experiment due to animal wellbeing concerns. Of those heifers that died or were removed due to BRD incidence, 7 were from the R15 treatment, 8 were from the R30 treatment, and 3 were from the R45 treatment. The remaining clinical health data are presented in Table 3.

**Table 3.** Effect of roughage inclusion in receiving diets on clinical health outcomes in newly received high-risk heifers (experiment 1)

	Experimental dietary treatment^1^				Contrasts^3^	
Variable	R15	R30	R45	SEM^2^	L	Q
Treated once for BRD^4^, %	14.17	15.74	11.40	4.372	0.53	0.44
Treated twice for BRD^5^, %	3.36	6.10	2.08	1.656	0.59	0.11
Treated thrice for BRD^6^, %	0.95	2.96	0.00	0.969	0.38	0.02
Total antimicrobial treatments^7^, %	18.07	24.40	13.00	6.245	0.44	0.13
Days to first BRD treatment	8.00	8.15	6.19	2.006	0.53	0.65
Rectal temperature^8^, °C	40.32	40.12	40.24	0.211	0.74	0.38
Severity score^9^	1.28	1.57	1.24	0.167	0.86	0.11

^1^Treatments included (DM basis): R15 =** **15% prairie hay, 46.50% Sweet Bran, 32.50% rolled corn, 6% dry supplement; R30 =** **30% prairie hay, 39% Sweet Bran, 25% rolled corn, 6% dry supplement; R45 =** **45% prairie hay, 31.50% Sweet Bran, 17.50% rolled corn, 6% dry supplement.

^2^ *N* = 9 pens per treatment.

^3^L = linear, Q = quadratic; *P*-value shown.

^4^Percentage of cattle treated once for bovine respiratory disease (BRD).

^5^Percentage of cattle treated twice for BRD.

^6^Percentage of cattle treated thrice for BRD.

^7^Total antimicrobial treatments for BRD were calculated by dividing the total number antimicrobial treatments administered within a pen by the sum of animals within the pen.

^8^Average rectal temperature of animals treated for BRD within a pen.

^9^Average severity scores of animals treated for BRD within a pen.

Throughout the experiment, there was no linear treatment response (*P* ≥ 0.38) on clinical health outcome variables. There was one quadratic response (*P* = 0.02) detected for the percent of animals treated three times; however, caution should be taken interpreting these results due to the low number of animals (*n* = 8) treated three times. Current dogma in the beef industry with regard to ration formulation for receiving calves is that morbidity and mortality increase as roughage inclusion decreases. Although no response existed for the total amount of animals treated for BRD in the current experiment, numerical differences and even a near-quadratic tendency for decreased morbidity as roughage level increases becomes apparent. Overall morbidity (13.8%) was less than expected (14.2%, 15.7%, and 11.4% for the R15, R30, and R45 diets, respectively; Table 3) based on the risk classification of the calves in the current experiment. The relatively low rates of morbidity observed may partially explain the lack in health differences observed between experimental treatments.

Similar trends in the response of morbidity to dietary treatment were observed between experiment 1 conducted by Lofgreen et al. (1975) and the current experiment, where both experiments reported that a greater percentage of animals consuming the intermediate diet required treatment for BRD compared to the low and high concentrate diets. Lofgreen et al. (1975) reported that calves were treated when rectal temperature exceeded 39.4 °C. In contrast, experiment 2 of Lofgreen et al. (1975), morbidity tended to increase with increasing concentrations of dietary energy. Richeson et al. (2019) suggested that calves enrolled on the high concentrate diet in experiment 2 of Lofgreen et al. (1975) experiment may have exhibited greater morbidity due to a reduced energy intake and subsequent inability to mount an adequate immune response, or that the calves may have experience ruminal acidosis. Energy intake for heifers was greatest on the R15 diet in the current experiment, which may describe why similar results in morbidity in this study were not observed. There are also possible limitations in interpreting the results from Lofgreen et al. (1975), as the authors used a small number of calves (range = 35 to 39) per treatment and only 2 pen replicates per treatment were used. The current experiment enrolled an average of 185 calves per experimental treatment and had 9 pen replications per treatment.

Fluharty and Loerch (1996) reported that no differences in calf morbidity existed between calves consuming diets containing 1.15, 1.21, 1.25, or 1.30 Mcal NE_g_/kg, and the authors concluded that calves may benefit from high energy receiving diets containing 70% to 85% concentrate. Diets in the current experiment contained 85%, 70%, and 55% concentrate for the R15, R30, and R45 diets, respectively. Again, while concentrate and roughage levels in the current experiment are similar to those reported throughout the literature, the proportion of that concentrate that is comprised of grain or the starch content of that concentrate varies between experiments. Approximately 30% to 40% of the concentrate in the diets would have come from starch in the current experiment. Regardless of differences in diet composition, the clinical results from the current experiment agree with results published by Fluharty and Loerch (1996).

Caution with interpretation of these morbidity data in the current experiment is warranted, however, due to the numerical increase observed (39.0% and 87.7% increase, respectively) in total antimicrobial treatments administered for BRD for calves consuming the R15 (18.07%) and R30 (24.40%) diets compared to calves consuming the R45 diet (13.00%) in the current experiment. Berry et al. (2004) also observed no differences in the percent of calves requiring BRD treatment when calves were assigned to 1 of 2 dietary energy levels (0.85 or 1.07 Mcal NE_g_/kg) and 1 of 2 dietary starch levels (34% or 48% of ME from starch). However, numerical differences were also observed in their experiment in which calves fed greater levels of starch had numerically increased morbidity, and calves consuming lower levels of starch tended to have less third antimicrobial treatments for BRD.

In the meta-analysis conducted by Rivera et al. (2005), the authors reported that morbidity attributed to BRD decreased slightly as dietary roughage concentration increased (% morbidity = 49.59 – 0.0675 × roughage %), but concluded that providing a 50% to 70% concentrate diet would be the optimum dietary strategy to enhance performance while negating negative effects on health in receiving calves. For the present experiment, linear increases in BW, ADG, and G:F were observed for calves receiving the 85% concentrate, 0.88 Mcal NE_g_/kg ration (R15), with no significant, negative effects on health being detected. Much of the foundational research concerning dietary energy density and roughage inclusion lacks the inclusion of fibrous byproducts that contribute to a large percentage of the diet in today’s feedlots (Samuelson et al., 2016; Richeson et al., 2019). Potentially, diets containing high energy, low starch fibrous byproducts may reduce instance of BRD without compromising performance. Lower starch, high-energy diets may also reduce the prevalence of digestive upsets such as clinical acidosis which can be difficult to distinguish from BRD clinically.

### Metabolite Characteristics (experiment 2)

**Figure 1.**
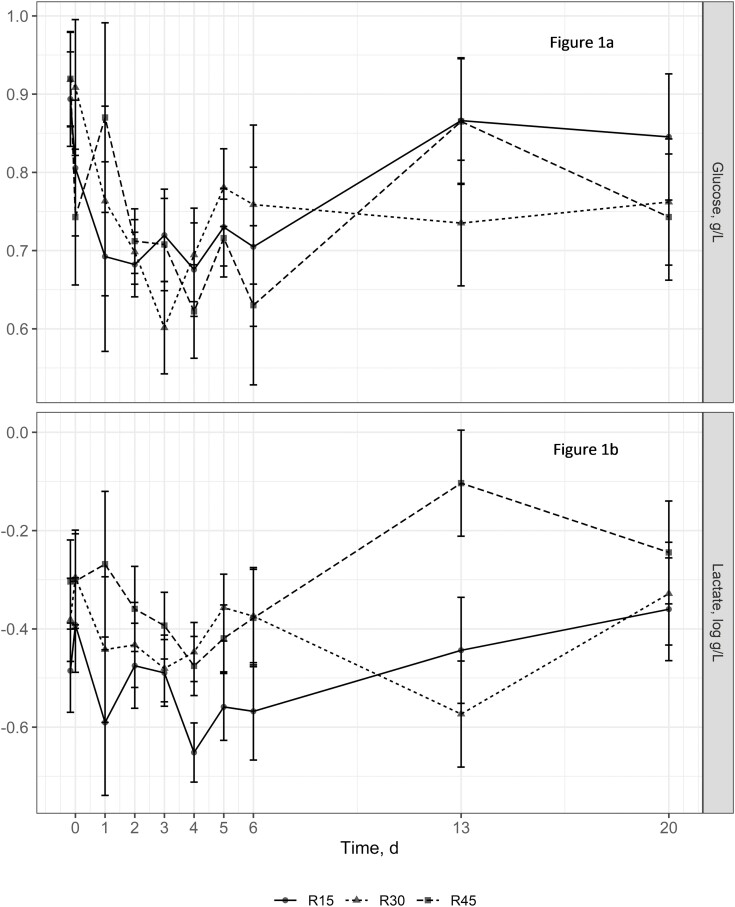
Effect of dietary treatment on serum glucose concentrations (a) and serum lactate (logarithm base 10) concentrations (b) in newly received beef calves (experiment 2). Treatments included (DM basis): R15 =** **15% prairie hay, 46.50% Sweet Bran, 32.50% rolled corn, 6% dry supplement; R30 =** **30% prairie hay, 39% Sweet Bran, 25% rolled corn, 6% dry supplement; R45 =** **45% prairie hay, 31.50% Sweet Bran, 17.50% rolled corn, 6% dry supplement. There was no treatment × time interaction (*P* = 0.92) or treatment effect (*P* = 0.96) on serum glucose concentrations. However, time did effect (*P* < 0.01) serum glucose concentrations. There was no treatment × time interaction (*P* = 0.45) or treatment effect (*P* = 0.11) for serum lactate concentrations. There was a time effect (*P* = 0.02) on serum lactate. Values plotted represent least squares means ± SE of the mean, calculated for 6 animals per experimental group.

**Figure 2.**
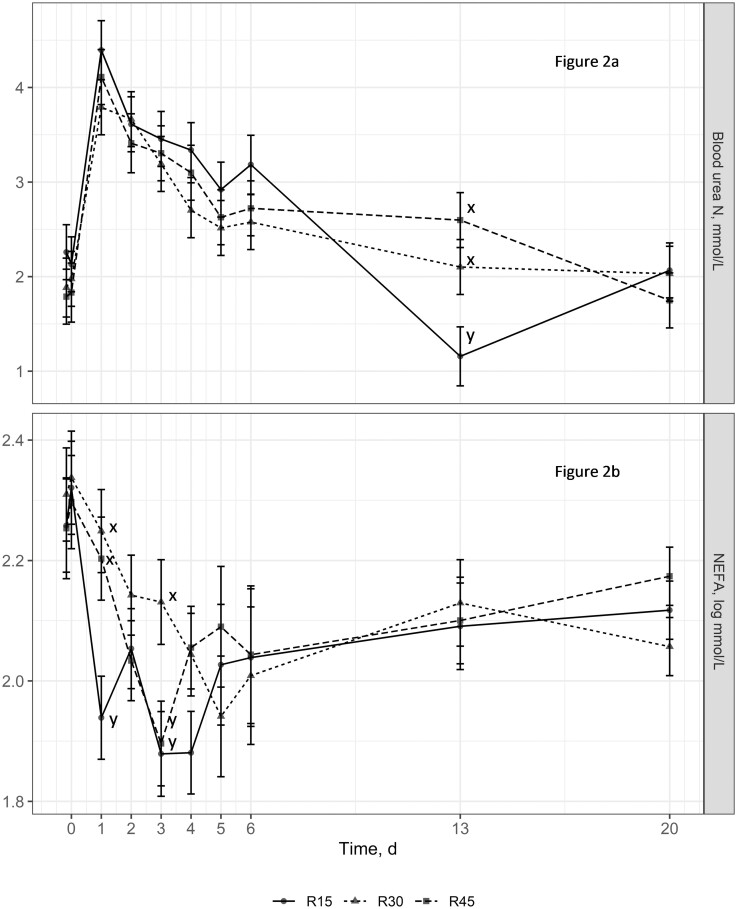
Effect of dietary treatment on blood urea nitrogen (BUN) concentrations (a) and nonesterified fatty acid (NEFA; logarithm base 10) concentrations (b) in newly received beef calves (experiment 2). Treatments included (DM basis): R15 =** **15% prairie hay, 46.50% Sweet Bran, 32.50% rolled corn, 6% dry supplement; R30 =** **30% prairie hay, 39% Sweet Bran, 25% rolled corn, 6% dry supplement; R45 =** **45% prairie hay, 31.50% Sweet Bran, 17.50% rolled corn, 6% dry supplement. There was a tendency for a treatment × time interaction (*P* = 0.07) for BUN concentrations. No treatment effect (*P* = 0.73) was observed for serum BUN concentrations; however, a time effect (*P* < 0.01) for serum BUN concentration was detected. There was a tendency for a treatment × time interaction (*P* = 0.06) for NEFA concentrations. No treatment effect (*P* = 0.47) was observed for serum NEFA concentrations; however, a time effect (*P* < 0.01) for serum NEFA was detected. Values plotted represent least squares means ± SE of the mean, calculated for 6 animals per experimental group. Within time points, the slice output option of SAS (SAS Inst. Inc., Cary, NC) was used to perform mean separations. ^x,y^Means with different superscripts tend to differ (*P* ≤ 0.10).

While animal performance measures were not analyzed in experiment 2 due to a lack of pen replication and the inability to measure individual animal DMI, summary statistics are presented in Table 4 to allow the reader to make inferences about the metabolite data. The summary statistics for performance in experiment 2 follow the same general trends as the performance data from experiment 1. Metabolite data from calves in experiment 2 are presented in Figures 1 and 2. There were no treatment × time interactions (*P* ≥ 0.45) observed for glucose or lactate concentrations. However, tendencies for treatment × time interactions were detected for BUN (*P* = 0.07) and NEFA (*P* = 0.06) concentrations. Concentrations of BUN tended to be greater for animals consuming the R30 and R45 diets on day 13 than for animals consuming the R15 diet. Nonesterified fatty acid concentrations tended to be greater on day 1 for animals consuming the R30 and R45 diets than for animals consuming the R15 diet. Additionally, NEFA concentrations were greater for the R30 experimental group than for the R15 and R45 treatment groups on day 3. The main effect of treatment did not impact (*P* ≥ 0.11) glucose and lactate concentrations; however, a time effect (*P* ≤ 0.02) was observed for all metabolites. Glucose concentrations decreased the first 2 d following exposure to dietary treatments. Concentrations of glucose consistently remained at around 0.6 to 0.7 g/L from day 2 to 4, then increased to day 5, decreased to day 6, and increased to day 13. Lactate concentrations decreased from days 0 to 4 followed by a slight increase to day 20. BUN concentration remained fairly steady from day 0 to h 4, increased substantially to day 1, then decreased over time to day 20. Blood NEFA concentrations decreased without a clear trend from hour 0 to day 4, which was then followed by a steady increase to day 20.

**Table 4.** Summary statistics: Effect of roughage inclusion in receiving diets on growth performance, intake, feed efficiency in newly received calves (experiment2)

	Experimental dietary treatment^1^		
Item	R15	R30	R45
BW^3^, kg			
d 0	268	267	264
d 21	303	296	292
ADG^4^, kg			
d 0 to 21	1.67	1.37	1.30
DMI^5^, kg			
d 0 to 21	6.39	7.46	7.79
G:F^6^			
d 0 to 21	0.262	0.183	0.167

^1^Treatments included (DM basis): R15 =** **15% prairie hay, 46.50% Sweet Bran, 32.50% rolled corn, 6% dry supplement; R30 =** **30% prairie hay, 39% Sweet Bran, 25% rolled corn, 6% dry supplement; R45 =** **45% prairie hay, 31.50% Sweet Bran, 17.50% rolled corn, 6% dry supplement.

^2^ *N* = 1 pen per treatment.

^3^Body weight was adjusted using a 2% calculated shrink.

^4^Pen ADG was calculated from individual shrunk BW gain, kg divided by days on feed.

^5^Pen DMI was calculated from total DMI for the pen for each period divided by the total calves and days on feed.

^6^Pen G:F was calculated by dividing the ADG for the pen by the average daily DMI for the pen.

The calves enrolled in experiment 2 were different from the heifers enrolled in experiment 1 in genetic makeup, previous management, and arrival physiological condition. Calves in experiment 2 were less stressed overall, as the calves were weaned, backgrounded, traveled less miles, were not commingled with calves from other sources, had previous vaccination records, and were clinically healthy at arrival. Therefore, the metabolite data presented should be used to assess dietary effects on metabolite characteristics in clinically healthy (low-risk; Wilson et al., 2017) receiving calves and should not be extrapolated to metabolite responses between dietary treatments in stressed high-risk calves subjected to shipping, commingling, pathogen exposure, and other stressful events during transition to the feedlot. Clinically healthy, low-risk calves were used in experiment 2 to elucidate the effects of the experimental dietary treatments on serum metabolites in a healthy metabolic state without interference from previous lack of nutrition, stress, or disease challenge.

Elevations in glucose concentrations have been caused by stresses and the resulting glucocorticoid production (Apple et al., 1995; Donley et al., 2009). Disruptions in glucose homeostasis due to immunoactivation and resulting increase in glucose consumption of immune cells have also been reported (Kvidera et al., 2017). Although no glucocorticoids such as cortisol were measured in experiment 2, it is plausible to speculate that the peak glucose concentrations measured on day 0 resulted from stresses incurred during shipping, feedlot entry, and from initial handling and processing. Glucose concentrations declined quickly after processing and began to increase again around day 4, which presumably is a function of increased feed intake during the first 4 d on feed.

In general, the trend in lactate concentration tended to follow that of glucose. Lactate is formed from glucose during anaerobic glycolysis under conditions of (local or systemic) hypoxia, including dehydration, maximum muscle exertion, compartment syndrome or other muscle injury, or severely compromised respiratory system. As such, it is not unexpected that lactate trends in the current experiment tended to follow those of glucose. The increased levels of lactate on day 0 are indicative of an increased stress response and subsequent switch of muscle metabolism to anaerobic from aerobic metabolism, which causes lactate to be transferred from the muscle and into the blood (Williams et al., 2019). No interaction or effect of treatment was detected for serum lactate. The reason for the numerical increase in lactate for the R45 treatment on day 13 is unknown.

BUN concentrations peaked 24 h following processing followed by a gradual decline throughout the remaining 20 d. BUN may reflect short-term dietary effects on rumen ammonia production and hepatic N turnover, which is plausible as the spike in BUN was 24 h following access to dietary treatments (Yari et al., 2010). These BUN data are indicative of an increased protein catabolism early in the receiving phase, and the resulting decrease following day 1 illustrates more reliance on dietary protein and less on muscle catabolism.

Serum NEFA concentrations are used to describe the extent of fat mobilization and are highly indicative of overall energy balance (Richeson et al., 2015; Buhler et al., 2019). Peak NEFA concentrations on day 0 likely reflect an overall negative energy balance as a result of the inherent stresses of transportation, receiving, and processing in combination with limited access to feed prior to processing. The subsequent decline in serum NEFA concentrations indicates that animals regained reliance on dietary energy as nutrients required to regain energy homeostasis were exceeded. The tendency for reduced NEFA concentrations on day 1 may be a result of a greater level of energy intake for calves consuming the R15 diet; however, an explanation for differences that tended to occur on day 3 is unknown. Collectively, minimal impacts of dietary treatments were observed on blood metabolites. It appears metabolite responses recorded in experiment 2 were more reflective of a typical transportation and processing procedures and time relative to feedlot arrival than dietary treatment. Further research should evaluate these responses to dietary treatments in high-risk calves subjected to additional stressors.

## CONCLUSIONS

Previous literature has indicated that performance, intake, and feed efficiency increase as dietary roughage decreases, but that improvements in performance come at the expense of slight increases in animal morbidity. Much of the previous research pertaining to roughage inclusion and diet energy density for receiving calves was conducted before the widespread use of fibrous byproducts that are commonly included in commercial feedlot diets today. Feeding a receiving diet containing 15% roughage and 0.88 Mcal NE_g_/kg in the current experiment provided superior performance without increasing the percentage of calves treated for BRD. It should be noted that overall morbidity in this experiment did not exceed 16% for any experimental treatment, so caution is warranted interpreting clinical outcomes, as morbidity results may differ between dietary treatments when a greater total percentage of calves become morbid. Body weight (BW), ADG, and G:F in the current experiment increased linearly while DMI decreased linearly as roughage inclusion decreased. Negligible differences were detected for serum glucose, lactate, BUN, or NEFA concentrations between dietary treatments; however, further research evaluating similar metabolites in stressed high-risk calves is perhaps warranted. Collectively the results of these experiments suggest that providing more energy dense receiving diets with lower levels of roughage may be a suitable alternative to traditional high roughage receiving diets when fibrous byproducts make up a large portion of the concentrate within the diet.
